# Signed in Blood: Circulating Tumor DNA in Cancer Diagnosis, Treatment and Screening

**DOI:** 10.3390/cancers13143600

**Published:** 2021-07-18

**Authors:** Jacob J. Adashek, Filip Janku, Razelle Kurzrock

**Affiliations:** 1 Department of Internal Medicine, University of South Florida, H. Lee Moffitt Cancer Center & Research Institute, Tampa, FL 33606, USA; 2 Department of Investigational Cancer Therapeutics (Phase 1 Clinical Trials Program), Division of Cancer Medicine, The University of Texas MD Anderson Cancer Center, Houston, TX 77030, USA; fjanku@mdanderson.org; 3 WIN Consortium, San Diego, CA 92093, USA

**Keywords:** ctDNA, next-generation sequencing, biomarkers

## Abstract

**Simple Summary:**

An important advance in the diagnostic and surveillance toolbox for oncologists is circulating tumor DNA (ctDNA). This technology can detect microscopic levels of cancer tissue before, during, or after treatment. Various groups from across the globe have published their experiences with the use of ctDNA to either guide therapy or monitor outcomes. The use of ctDNA likely cannot supplant the need for tissue biopsies, but it can complement other diagnostic and therapeutic monitoring mechanisms.

**Abstract:**

With the addition of molecular testing to the oncologist’s diagnostic toolbox, patients have benefitted from the successes of gene- and immune-directed therapies. These therapies are often most effective when administered to the subset of malignancies harboring the target identified by molecular testing. An important advance in the application of molecular testing is the liquid biopsy, wherein circulating tumor DNA (ctDNA) is analyzed for point mutations, copy number alterations, and amplifications by polymerase chain reaction (PCR) and/or next-generation sequencing (NGS). The advantages of evaluating ctDNA over tissue DNA include (i) ctDNA requires only a tube of blood, rather than an invasive biopsy, (ii) ctDNA can plausibly reflect DNA shedding from multiple metastatic sites while tissue DNA reflects only the piece of tissue biopsied, and (iii) dynamic changes in ctDNA during therapy can be easily followed with repeat blood draws. Tissue biopsies allow comprehensive assessment of DNA, RNA, and protein expression in the tumor and its microenvironment as well as functional assays; however, tumor tissue acquisition is costly with a risk of complications. Herein, we review the ways in which ctDNA assessment can be leveraged to understand the dynamic changes of molecular landscape in cancers.

## 1. Introduction

A liquid biopsy is a minimally invasive technique for measuring diagnostically significant tumor-derived markers in body fluids. Although any liquid can be biopsied (e.g., blood, urine, ascites, and cerebrospinal fluid), herein we will be referring to blood biopsies when we speak of liquid biopsies. The types of components that can be interrogated in a liquid biopsy include circulating tumor cells, circulating extracellular nucleic acids (cell-free DNA (cfDNA) and its neoplastic fraction—circulating tumor DNA (ctDNA)), as well as extracellular vesicles (such as exosomes), and a variety of glycoproteins. We will be focused on ctDNA and cfDNA. cfDNA is a broad term that refers to DNA which is freely circulating in the blood but is not necessarily of tumor origin [1]; ctDNA is fragmented DNA in the bloodstream that is of tumor origin and is not associated with cells.

**Figure 1:**
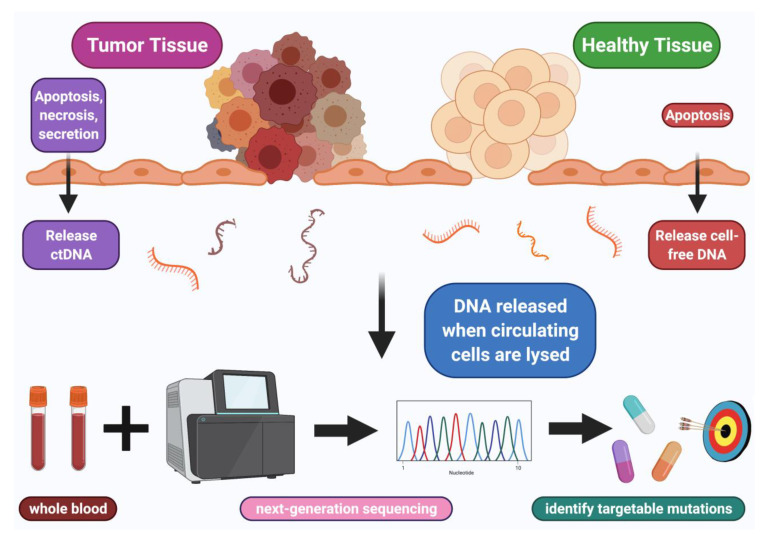
ctDNA employed to identify targetable mutations. Blood sample collected and next-generation sequencing performed on shed ctDNA from tumor, which can identify genomic alterations, some of which may be pharmacologically tractable.

The use of next-generation sequencing (NGS) of ctDNA from a blood biopsy has gone, in the last decade, from the unimaginable to the routine. NGS of ctDNA has provided insights into potential genomic-derived treatment options such as identifying novel targets as well as predicting responses to treatments (Figure 1) [2].

Liquid biopsies can also be used to evaluate microsatellite stability/instability (MSI-H) and high tumor mutational burden (TMB-H), both of which are critical parameters for predicting immune checkpoint blockade response [3,4,5]. Further, ctDNA can be exploited to monitor response and predict resistance in some tumors [6,7,8,9,10].

The half-life of ctDNA ranges from 30 min to two hours. Changes in ctDNA can be used to monitor tumors dynamically [11]. Both the concentration of ctDNA and the number of somatic alterations found within a sample have been implicated in some studies as a surrogate for tumor stage and size as well as tumor aggressiveness [12,13,14].

The implementation of diagnostics using ctDNA has been leveraged as a companion diagnostic test, e.g., for detecting EGFR inhibitor sensitive mutations for the use of erlotinib in non-small cell lung cancer [15]. Even so, ctDNA may provide important risk stratification data [16].

A challenge for the utility of ctDNA is that clonal hematopoiesis of indeterminate potential (CHIP) may confound results; in other words, some presumptive ctDNA mutants may be derived from aberrations in blood cells, particularly those that accompany aging, rather than abnormalities in the tumor, yet mutational burden from CH is low and can be excluded by sequencing healthy control tissue [17].

Herein, we will examine the multiple potential uses of liquid biopsy with NGS of ctDNA in oncology:

○ early diagnosis of cancer;
○ ctDNA as a prognostic variable;
○ measurement of residual disease;
○ discerning molecular alterations that can inform therapeutic decision-making; and
○ monitoring response, resistance, and burden/aggressiveness of disease.

## 2. Comparison of CTCs, ctDNA, and Tissue DNA

Circulating tumor cells (CTCs), ctDNA, and tissue DNA (tDNA) are all potentially exploitable for providing insight and data about tumor genomes (Table 1). Acquisition of CTCs and ctDNA are both noninvasive, requiring only a venipuncture, and are considered to be liquid biopsies. In contrast, tissue DNA requires an invasive biopsy.

CTCs are tumor cells that are shed from growing and dying tumors that require isolation; thus, they technically require specialized equipment. However, CTCs can be a rich source of information about the genomic, transcriptomic, and proteomic content of the tumor; if grown in culture, CTCs can also provide functional assays. In contrast, ctDNA, while being easier to isolate that CTCs, cannot be cultured and the information obtainable from ctDNA is generally restricted to genomic analysis [18]. In this regard, blood-derived CTCs and tissue samples share similarities, as both can be isolated, cultured, and provide genomic, transcriptomic, and proteomic tumor data.

In a blood sample (~10 cc), there will be tens to hundreds of ctDNA fragments for testing, whereas there will likely only be a handful of CTCs.

One limitation of tissue DNA is that it is obtained from a discrete piece of tumor tissue; thus, tissue DNA cannot reflect heterogeneity amongst metastatic sites and is more difficult to be followed serially [19]. CTCs and ctDNA are, however, shed from multiple metastatic sites and therefore better reflect tissue heterogeneity than a tissue biopsy. On the other hand, the requirement for extremely sensitive techniques for genomic interrogation of CTCs and ctDNA means that tissue assays often yield greater numbers of positive genomic alterations, and tissue NGS assays are generally more comprehensive than those applied to ctDNA.

A unique advantage to CTCs and ctDNA is their amenability to longitudinal follow up with a simple blood test in order to predict therapeutic response and resistance [19].

## 3. Liquid Biopsy and Dynamics of Normal Versus Tumor Cell-Free DNA (cfDNA)

Elevated levels of cfDNA were found in patients with cancers but can be detected during pregnancy and in patients with history of organ transplant [20]. Generally, the blood concentration of cfDNA can vary from 0–5 to >1000 ng/mL in cancer patients and between 0 and 100 ng/mL in otherwise healthy patients [21,22]. The large range of cfDNA and ctDNA found in patients with cancer is in part due to the fact that various tumor types can have wide variations in ctDNA shedding and that the amount of ctDNA can reflect tumor burden. Patients with brain, kidney, and thyroid cancers have been found to have lower levels of cfDNA than those patients with pancreatic, colorectal, ovarian, breast, gastroesophageal, and melanoma [13,23]. Additionally, premalignant and early-stage cancers generally have lower levels of cfDNA compared to patients with advanced disease [21].

Not all of the cfDNA in the bloodstream of cancer patients is ctDNA, and it is important to recognize what fraction of cfDNA is actually from a cancer. It is believed that ~0.1–89% of cfDNA is made up of ctDNA and that the ratio may increase as a cancer progresses [13,24,25]. The sizes of cfDNA are estimated to be between 40 and 200 base pairs [26,27,28]. If wrapped in chromatin, the DNA in these vesicles can make up to 2 million base pairs [29]. These fragments are believed to be part of tumor metabolism and growth; fragments from necrotic tumor tissue can be over 10,000 kilobases [30].

The amount of cfDNA found within the bloodstream is dependent on the balance of release and clearance of cfDNA. Clearance can occur within the primary tumor tissue, within the blood, or within various filtration organs: spleen, liver, and lymph nodes [31]. Elevated levels of cfDNA in patients with cancer is believed to be in part because of lack of clearance and subsequent accumulation. Within the bloodstream, degradation of cfDNA is performed in large part by circulating enzymes: deoxyribonuclease (DNAse) I, plasma factor VII-activating protease, and factor H [32,33]. Within the spleen and liver, Kupffer cells and macrophages have been implicated in removing cfDNA and nucleosomes from circulation [34]. The presence of tumor in patients with cancer may be in part the reason for higher levels of cfDNA detected and also in part due to inability to clear these fragments within these various mechanisms.

## 4. How ctDNA Enters and Leaves the Circulation

It is unclear exactly how ctDNA enters the blood stream; however, it is postulated that when primary tumor cells or metastatic cells die via apoptosis or necrosis, DNA fragments may be released into the bloodstream [22,35]. The amount of ctDNA that can be found within the blood stream is heavily dependent on the overall tumor biology and burden. The half-life of ctDNA is estimated to be between 30 min and two hours; ctDNA is rapidly degraded by bloodstream DNases [31].

## 5. Technological Methods for cfDNA Extraction and Sequencing

Various techniques to detect ctDNA are available all with varying advantages and limitations (Table 2). These techniques include droplet digital polymerase chain reaction (ddPCR), beads, emulsion, amplification, and magnetics (BEAMing), tagged-amplicon deep sequencing (TAm-Seq), cancer personalized profiling by deep sequencing (CAPP-Seq), whole exome sequencing (WES), and whole genome sequencing (WGS).

ddPCR can identify potentially rare mutations, calculate copy number variants, as well as inform on miRNA [36]. This method also allows for detection of very low levels of genomic material, 0.01–1.0% [37]. The most notable limitation of this method of ctDNA detection, however; is that only characterized sequences can be screened via this method.

The use of BEAMing allows for the assessment of characterized alterations (e.g., SNVs, indels, and amplifications) and combines PCR with flow cytometry [43]. This allows for the detection of alterations at exceedingly low levels—0.01%—with marked concordance to tissue testing of 91.8% [38]. 

The CAPP-Seq technique utilizes large genomic libraries combined with individual patient sample sequence signatures to identify alterations within ctDNA. This method combines statistical assessment of well-characterized tumor alterations with DNA oligonucleotides to identify patient specific alterations [39]. This method allows for the identification of various genomic alterations such as insertions/deletions, single nucleotide variants, rearrangements, and copy variants. A limitation of CAPP-Seq includes the inability to identify fusions, in contrast to ddPCR, TAm-Seq, WES, and WGS [39].

The TAm-Seq technique allows for highly sensitive and specific analysis ~97% along with the ability to detect low levels of ctDNA, 2%. This method uses primers to tag and identify the desired genomic sequence. The limitation with this technique is that the sequence needs to be characterized to be included in the analysis [40].

Whole exome sequencing allows for comprehensive analysis and characterization of potentially all tumor mutations. In doing so, the sensitivity may be lower than other modalities because it includes all exomic alterations. The limitations of WES relate to error rate and sensitivity [41,44].

WGS includes the entire tumor genome to discern characterized/deleterious alterations as well as many uncharacterized genomic events (variants of uncertain significance (VUSs) and is mainly used for CNAs [42].

## 6. Clinical Laboratory Improvement Amendments (CLIA) Grade Commercially Available ctDNA Assays

There are several CLIA-grade commercially available ctDNA assays that clinicians can order to potentially inform treatment decisions for patients. One of the most widely available of these tests is the Guardant360 CDx from Guardant Health, which was first accessible in 2014. The Guardant360 assay includes 73 genes commonly altered in cancers and can identify single-nucleotide variants (SNVs), insertions/deletions (indels), fusions, and copy number alterations (CNAs) (https://www.therapyselect.de/sites/default/files/downloads/guardant360/guardant360_specification-sheet_en.pdf, accessed date: 10 January 2021). This Guardant360 assay requires two 10 cc tubes of whole blood and is reported to have results in 7 calendar days after receipt of the samples.

In 2018, Foundation Medicine released their ctDNA assay called FoundationOne Liquid, which now includes 311 genes implicated in cancers (https://assets.ctfassets.net/w98cd481qyp0/wVEm7VtICYR0sT5C1VbU7/55b945602d7dc78f42b3306ca1caa451/FoundationOne_Liquid_CDx_Technical_Specifications.pdf, accessed date: 10 January 2021). The FoundationOne Liquid assay included base substitutions, indels, rearrangements, copy number alterations, and MSI-H status. The FoundationOne Liquid assay requires two 8.5 cc tubes of whole blood and reports to have results within less than two weeks after receipt of the samples.

Also in 2018, Tempus introduced Tempus xF, a ctDNA assay, which includes 105 genes implicated in cancers. The Tempus xF assay included SNVs, indels, rearrangements/fusions, CNAs, and MSI-H status (https://www.tempus.com/wp-content/uploads/2020/02/xF-Validation_013020-2.pdf, accessed date: 10 January 2021). The Tempus xF assay requires two 8 cc tubes of whole blood and reports to have results in nine to 14 days after receipt of the samples.

## 7. Food and Drug Administration (FDA) Approvals for ctDNA Tests

In August 2020, the FDA approved the use of FoundationOne Liquid CDx test from Foundation Medicine, Inc. as a companion diagnostic test for patients with ovarian cancer to identify mutations in *BRCA1/2* for the use of rucaparib, for patients with metastatic hormone-resistant prostate cancer with mutations in *BRCA1/2* and *ATM* for the use of olaparib, for patients with metastatic hormone-resistant prostate cancer with mutations in *BRCA1/2* for the use of rucaparib, for patients with non-small cell lung cancer (NSCLC) with *ALK* rearrangement for the use of alectinib, for patients with NSCLC with *EGFR* exon 19 deletions and *EGFR* exon 21 L858R alterations for the use of gefinitinb, erlotinib, and osimertinib, and for patients with breast cancer with mutations in *PIK3CA C420R, E542K, E545A, E545D [1635G > T only], E545G, E545K, Q546E, Q546R, H1047L, H1047R, and H1047Y* for the use of alpelisib [45,46].

Guardant360 CDx by Guardant Health Inc. was also approved in 2020 to identify *EGFR* exon 19 deletions, L858R, and T790M mutations in patients with NSCLC for the use of Osimertinib [47].

The Therascreen PIK3CA RGQ PCR Kit was approved in 2019 to detect 11 mutations in the PIK3CA gene in patients with metastatic breast cancer for the use of alpelisib [48].

Additionally, Cobas EGFR Mutation Test v2 was also approved in 2016 to identify *EGFR L858R* mutations in patients with NSCLC for the use of erlotinib [49].

## 8. Clinical Uses of ctDNA

### 8.1. ctDNA for Early Diagnosis of Cancer

Early cancer detection could transform outcomes by detecting lethal tumors at a time when the malignancies are curable, and treatment invokes less morbidity. However, the technical, biological, and clinical hurdles to developing an effective pan-cancer screening test for early cancer are substantial.

Liquid biopsies with NGS of ctDNA are an attractive tool, but the very small amounts of ctDNA in early disease is still a major technical challenge, as is the issue that non-cancerous normal tissue may have somatic mutations indistinguishable from those in cancer, but as mentioned above, CH mutations can be filtered out by using healthy tissue control samples. Still, Cohen et al. developed a noninvasive blood test, called CancerSEEK, that detected eight common cancer types through assessment of circulating proteins and mutations in cfDNA. In a study of 1005 patients previously diagnosed with non-metastatic cancer and 850 healthy control individuals, CancerSEEK detected cancer with a 99% specificity and a sensitivity of 69% to 98% (depending on type of malignancy) [50].

Another methodology that has recently been exploited is assessing ctDNA methylation patterns, noting that increased methylation of tumor suppressor genes can be seen as an early inciting event in the carcinogenesis of various tumors, such as hepatocellular and colorectal carcinomas [51]. A prospective case–control study evaluated the performance of pan-cancer targeted methylation analysis of cfDNA. With 6689 participants (2482 cancers (>50 cancer types), 4207 healthy), specificity was 99.3% and stage I–III sensitivity was 43.9% in all cancer types [52]. Other unique technologies aimed at early cancer detection continue to be explored.

### 8.2. ctDNA as a Prognostic Variable

To date, multiple studies have analyzed the utility of ctDNA to be able to assess disease-free survival (DFS) and overall survival (OS) (Table 3) [53,54,55,56,57,58,59,60,61,62,63,64,65,66,67]. Factors that predict poorer outcomes include concordance between tissue and liquid ctDNA alterations (shown for both *TP53* and *KRAS* mutations) [68,69], higher percent ctDNA (perhaps reflecting higher tumor burden, and higher number of ctDNA alterations [14].

Multiple studies have shown that ctDNA can be an important prognostic factor. For instance, in triple-negative breast cancer patients who had received or were receiving neoadjuvant chemotherapy, the detection of ctDNA was associated with a significantly worse DFS (*p* = 0.027) [53]. Additionally, at the last post-chemotherapy pre-surgery time point, detection of ctDNA was strongly associated with shorter DFS (*p* = 0.013) and OS (*p* = 0.006) [53]. In patients receiving adjuvant chemotherapy for locally advanced rectal cancer, 122 (77%) of 159 patients had pre-surgical detectable ctDNA and after surgery only 12 of 140 (8.6%) with negative ctDNA (hazard ratio (HR) 12, *p* < 0.001) experienced recurrence [54]. Further, post-op ctDNA detection predicted recurrence regardless of adjuvant chemotherapy (chemotherapy: HR 10, *p* < 0.001; no chemotherapy: HR 16, *p* < 0.001) and ctDNA detection predicted higher recurrence rate among patients with a pathological complete response (HR 14, *p* = 0.014) or with pathologic node-positive disease (HR 11, *p* < 0.001) [54]. A cohort study of patients with local advanced anal squamous cell cancer found that, in 33 patients, ctDNA detection after chemoradiation was associated with shorter DFS (*p* < 0.0001) [55]. Additionally, this study reported that ctDNA was associated with stage (64% in stage II and 100% in stage III; *p* = 0.008) and baseline ctDNA levels were higher in pathological node positive (median 85 copies/mL, range = 8–9333) than pathological node negative disease (median 32 copies/mL, range = 3–1350) *p* = 0.03 [55]. In another study, this one in pancreatic cancer, higher levels of total %ctDNA were an independent prognostic factor for worse survival (hazard ratio, 4.35; 95% confidence interval, 1.85–10.24 (multivariate, *p* = 0.001)) [63].

### 8.3. ctDNA to Measure Residual Disease

The ability of ctDNA to track tumor-specific mutations and to detect occult cancer lend themselves naturally to assessment of minimal residual disease. Further, the ease of plasma sampling permits ctDNA levels to be serially followed in order to longitudinally trend mutation status and frequently assess dynamic changes in levels of ctDNA, as reflected by percent ctDNA (or variant allele fraction (VAF).

Multiple studies are now beginning to confirm clinical utility of ctDNA in evaluating minimal residual disease [73]. For instance, declines in circulating allele fractions of relevant mutations have been associated with clinical outcomes in melanoma, colorectal cancer, breast and ovarian cancer, and EGFR-positive lung cancer [74,75,76,77,78]. As examples, in a study that monitored patients with colorectal cancer pre- and post-surgery, pretreatment ctDNA was detected in 93.4% (100/107) of patients; post-operative ctDNA status was assessed in 107 patients, of whom, 13% (14/107) were minimal residual disease-positive. Of the positive patients, 42.9% (6/14) eventually relapsed while only 8.6% (8/93) of the negative patients relapsed (HR: 10; 95% CI: 3.3–30; *p* < 0.001). In multivariate analysis, ctDNA status was the most significant prognostic factor associated with relapse-free survival (HR: 28.8, 95% CI: 3.5–234.1; *p* < 0.001) [79]. Similarly, in patients undergoing surgery for peritoneal metastases, high levels of pre-operative ctDNA and new postoperative ctDNA alterations in the context of preoperative alterations predicted worse outcomes [59]. Applications may include using ctDNA to determine escalation or de-escalation of adjuvant therapy.

### 8.4. Discerning ctDNA Molecular Alterations That Can Inform Decision Making

Multiple studies demonstrate the important use of ctDNA interrogation for prosecuting treatment. In fact, as mentioned above, the FDA has approved several ctDNA tests as companion diagnostics [45,46,47]: detection of *BRCA1/2* alterations for the use of the poly (ADP-ribose) polymerase (PARP) inhibitor rucaparib in ovarian cancer; *BRCA1/2* and *ATM* mutations for the use of the PARP inhibitor olaparib in prostate cancer; *ALK* and *EGFR* alterations to be treated with the ALK inhibitor alectinib or the EGFR inhibitors gefitinib, erlotinib, and osimertinib in NSCLC; and a variety of *PIK3CA* alterations to be treated with the PIK3CA inhibitor alpelisib in breast cancer.

Numerous other studies support the utility of ctDNA for genomic characterization aimed at assisting therapeutic choice. For instance, one study in patients with advanced breast cancer found that 68% (42/62) of patients had ≥1 characterized/pathogenic ctDNA alteration (non-VUS) [57]. A similar study in patients with advanced and resected esophageal, gastroesophageal junction, and gastric adenocarcinoma found that 76% (42/55) of patients had a ctDNA alteration, with 69% (38/55) having ≥1 characterized/deleterious (non-VUS) [58]. In gynecologic cancers, therapy matched to ctDNA alterations (*n* = 33 patients) was independently associated with improved survival (HR: 0.34, *p* = 0.007) compared to unmatched therapy (*n* = 28 patients) in multivariate analysis [60]. In a study focused on *EGFR* amplification, such amplifications were detected in cfDNA in a significant subset of pan-cancer patients—8.5% of 28,584. Most patients had coexisting alterations. Importantly, responses were observed in five of nine patients who received EGFR inhibitors, including patients who showed ctDNA EGFR amplifications, but no amplifications in the tissue DNA [64]. Taken together, it is apparent that ctDNA molecular alterations play a vital 79 Burden/Aggressiveness of Disease

Resistant ctDNA alterations that may emerge months before changes in scans are noted and can inform an understanding of mechanisms of resistance in colorectal, lung, and breast cancers, as examples [80,81,82]. For instance, ctDNA was used to identify early resistance mutations in patients with *HER2*-amplified breast cancer; PI3K/mTOR pathway alterations were the major cause of resistance [83]. This information may be exploitable with the addition of another targeted therapeutic [84]. Using ctDNA in a longitudinal fashion could allow for concomitant or sequential targeting of multiple gene mutations in real-time. This strategy and the ability of ctDNA to offer this information prior to imaging and without the need for additional tissue biopsies may be part of the holy grail of getting the right drug to the right patient at the right time. Based on criteria established by the OncoKB database, and other evidential reports, studies have shown that over one-quarter of cancers harbored level 1 actionable targets in their ctDNA [85]. The ability to find these mutations early in the treatment course could potentially alter the trajectory of recognizing mutation acquisition, thus enhancing patient outcomes.

Furthermore, ctDNA can be an early marker of response. For instance, drug-induced tumor apoptosis may occur for EGFR-targeted therapy in lung cancer within days of initial dosing, and daily sampling of ctDNA may facilitate early assessment of patient response within the first week of treatment with EGFR inhibitors [10]. Similarly, ctDNA has been used to predict response to treatment before radiographic response in colorectal cancer [75]. This measurable entity portends survival even in the setting of neoadjuvant therapy of breast cancer [86].

Similarly, early plasma ctDNA changes predicted response to first-line pembrolizumab in in patients with lung cancer [70]. Finally, genome-wide sequencing of cfDNA identified copy number alterations that could be used for monitoring early response (or resistance) to immunotherapy in cancer patients [72,87].

Multiple publications also show that both %ctDNA (VAF) and number of alterations in ctDNA predict a poor prognosis, possibly because they reflect tumor burden and/or aggressiveness [14].

## 9. The Issue of Concordance between ctDNA and Tissue DNA

Several studies have examined the concordance in molecular alterations between tissue and ctDNA samples. In general, concordance is variable ranging from ~50% to over 95% [69,88]. The literature suggests that the results from liquid biopsies and from tissue biopsies, vis a vis NGS, are highly reproducible [89]. Therefore, biological differences most likely account for discrepant ctDNA and tissue DNA NGS results.

The biologic attributes that underlie differences between tissue and ctDNA results include (i) shedding of DNA into the bloodstream may be limited from some sites, (ii) ctDNA can be suppressed by treatment, and (iii) tissue DNA tests the genomics in a small sample of tissue, whereas ctDNA may reflect shed DNA from multiple metastatic sites. Both tissue and ctDNA may be confounded by germline alterations and by clonal hematopoiesis of indeterminate potential, though ctDNA may be more vulnerable to such confounders.

Interestingly, studies now show that concordance between ctDNA and tissue DNA alterations, at least for *TP53* and for *KRAS,* is associated with worse outcomes [68,69].

## 10. Conclusions and Future Directions

Tumors release ctDNA into the bloodstream. The amount of ctDNA discernable, as reflected by percent of DNA VAF and the number of ctDNA alterations, may be an indicator of tumor burden and/or aggressiveness, with higher numbers predicting worse prognosis.

Blood-derived ctDNA may provide crucial molecular information as a complement to the tumor biopsy for the following reasons: (i) some cancer tissue is not easily or safely accessible for biopsy; (ii) even if accessible, tumor biopsies can be complex and expensive procedures with morbidity; (iii) over time, the tissue that was biopsied may become less representative of the tumor, since malignancies undergo genomic evolution; (iv) genomic aberrations discerned in a tissue biopsy reflect the content of the small tissue sample, while ctDNA NGS abnormalities may reflect the heterogeneous alterations found in shed DNA from many metastatic sites; and (v) dynamic changes in ctDNA can occur and reflect response or resistance to treatment. Furthermore, evaluating ctDNA pre- or post-surgery may serve as a predictive tool for recurrence risk. Finally, ctDNA may be exploitable for early detection of lethal cancers when they are still curable and/or do not require drastic, life-altering interventions.

There are also disadvantages to ctDNA as compared to tissue DNA assessment: (i) ctDNA is found in only small amounts in the circulation, making it difficult to detect alterations, and (ii) ctDNA carrying tumor-specific alterations may represent only a small fraction of the total genomic alterations in the tumor, since not all cancer-derived DNA may be shed into the blood. Therefore, variability in concordance rates between blood-derived ctDNA samples and tissue samples can be caused by spatial and temporal variables, as well as by dynamic changes driven by therapy and disease evolution; and (iii) ctDNA is more liable to be confounded by alterations of clonal hematopoiesis of indeterminate potential, and perhaps also by germline alterations.

Taken together, the literature indicates that assessment of blood-derived ctDNA is a powerful and transformative technology which can inform genomic decision making for gene- and immune-targeted therapy, can predict prognosis, and can be followed serially to assess response, resistance, and residual disease.

## Figures and Tables

**Table 1:** Comparison of CTCs, circulating tumor DNA (ctDNA), and tissue DNA (tDNA).

	Circulating Tumor Cells (CTC)	Circulating Tumor DNA	Tissue DNA
**Able to be cultured**	Able to be cultured	Unable to be cultured	Unable to be cultured
**Ability to assess genomic, transcriptomic, proteomic data**	Able to assess DNA, RNA and protein	Only able to assess DNA	Able to assess DNA, RNA, protein and tumor-infiltrating lymphocytes
**Influences of collection and interpretation**	Potential for sample bias	Minute amounts ctDNA in blood stream	Sample can be from primary or metastatic lesions
**Ability to predict therapy responses**	Serial samples can be predictive of responses to therapy	Serial samples can be predictive of responses to therapy	Serial samples are invasive and have not been shown to be a predictor of response, though new genomic alterations may predict resistance
**Modifying factors**	Heterogeneity within shed cells can be considered an opportunity as tissue biopsies might miss specific clones based on the location/size of the piece of tumor that was taken for analysis	Rate of tumor cell apoptosis, necrosis, and clearance. Possibly size of tumor sites and possibly number and location of metastatic sites can impact ctDNA levels	Tumor heterogeneity within primary and between primary and metastatic sites can occur

The bold means 5 distinct comparing/contrasting items that are not related.

**Table 2:** Examples of techniques to detect ctDNA.

Technique	Advantages	Limitations	References
Droplet digital PCR (ddPCR)	High sensitivity	Only detects specific genomic sequences within sample	[36,37]
Beads, emulsion, amplification and magnetics (BEAMing)	High sensitivity	Only detects known alterations	[38]
Cancer Personalized Profiling by deep Sequencing (CAPP-Seq)	High sensitivity	Not fully comprehensive	[39]
Tagged-amplicon deep sequencing (TAm-Seq)	High sensitivity	Not fully comprehensive	[40]
Whole exome sequencing (WES)	Includes entire exome	Lower sensitivity	[41]
Whole genome sequencing (WGS)	Includes entire genome	Lower sensitivity	[42]

**Table 3:** Examples of studies investigating clinical applications of ctDNA.

Cancer Histology	Setting	Results	References
Triple-negative breast cancer	During/after neoadjuvant chemotherapy	After cycle 1, detection of ctDNA was associated with worse DFS (*p* = 0.027) At the last post-chemotherapy pre-surgery time point, detection of ctDNA was strongly associated with worse pCR and DFS (*p* = 0.013) and OS (*p* = 0.006)	[53]
Advanced breast cancer	Therapeutic planning and serial testing for treatment response, tumor genomic evolution detection	68% (42/62) of patients had ≥1 characterized ctDNA alteration (non-VUS) Concordance between tDNA and ctDNA was 48%	[57]
Ovarian, uterine, cervical, vulvovaginal, and unknown gynecologic primary	Therapeutic planning and serial testing for treatment response, tumor genomic evolution detection	Therapy matched to ctDNA alterations (*n* = 33) was associated with improved OS (HR: 0.34, *p* = 0.007)	[60]
Locally advanced rectal cancer	Adjuvant chemotherapy	122 patients had pre-surgical detectable ctDNA Only 12 of 140 (8.6%) with negative ctDNA (HR 12, *p* < 0.001) experience recurrence Post-op ctDNA detection predicted recurrence regardless of adjuvant chemotherapy (chemo: HR 10, *p* < 0.001; no chemo: HR 16, *p* < 0.001) ctDNA detection predicted recurrence among pts with a pCR (HR 14, *p* = 0.014) or with pN+ disease (HR 11, *p* < 0.001)	[54]
Local advanced anal squamous cell cancer	Prognostic impact of post chemoradiation ctDNA detection	ctDNA detection after chemoradiation was associated with shorter DFS (*p* < 0.0001) More ctDNA was associated with higher stage (64% in stage II and 100% in stage III; *p* = 0.008) baseline ctDNA levels were higher in pN+ (median 85 copies/mL, range = 8–9333) than pN- (median 32 copies/mL, range = 3–1350) *p* = 0.03	[55]
Advanced colorectal cancer	Therapeutic planning and serial testing for treatment response, tumor genomic evolution detection	81% (63/78) of patients had ctDNA alteration, with 76% (59/78) having ≥1 characterized (non-VUS) Concordance between tDNA and ctDNA ranged from 62–87%	[56]
Biliary tract cancers	Therapeutic planning and serial testing for treatment response, tumor genomic evolution detection	40 patients with both ctDNA and tDNA sequencing, concordance was higher between ctDNA and metastatic site tDNA than between ctDNA and primary tDNA (78% vs. 65% for TP53, 100% vs. 74% for KRAS and 100% vs. 87% for PIK3CA Therapy matched to genomic alterations (*n* = 80) had significantly longer PFS (HR 0.60, CI 0.37–0.99; *p* = 0.047) and higher disease control rate (61% vs. 35%; *p* = 0.04)	[61]
Advanced and resected esophageal, GEJ, and gastric adenocarcinoma	Therapeutic planning and serial testing for treatment response, tumor genomic evolution detection	76% (42/55) of patients had ctDNA alteration, with 69% (38/55) having ≥1 characterized (non-VUS) Concordance between tDNA and ctDNA ranged from 61 to 87%	[58]
Advanced pancreatic ductal adenocarcinoma	Therapeutic planning and serial testing for treatment response, tumor genomic evolution detection	Concordance between ctDNA and tDNA for TP53 was 61% and for KRAS 52% Concordance for KRAS between ctDNA and tDNA from metastatic sites was significantly higher than between ctDNA and primary tDNA (72% vs. 39%, *p* = 0.01) Higher levels of total %ctDNA was associated with worse survival (HR, 4.35, CI 1.85–10.24; *p* = 0.001)	[63]
Advanced NSCLC	Changes in VAF were serially measured in patients receiving pembrolizumab and platinum doublet-chemotherapy	VAF decreased by 90.1% at median 21 days after treatment in patients (*n* = 18) with radiographic response VAF decreased by 19.9% in patient (*n* = 15) with stable disease (*n* = 15) VAF increased by 28.8% in patients (*n* = 12) with progressive disease; *p* = 0.003 VAF decrease between the pretreatment and first on-treatment blood draw was associated with higher ORR (60.7% vs. 5.8%; *p* = 0.0003), VAF decrease between the pretreatment and first on-treatment blood draw was associated longer median PFS (8.3 vs. 3.4 months, HR: 0.29, CI 0.14 to 0.60; *p* = 0.0007) VAF decrease between the pretreatment and first on-treatment blood draw was associated longer median OS (26.2 vs. 13.2 months, HR: 0.34, 0.15 to 0.75; *p* = 0.008	[70]
Advanced lung cancers	Ultra-deep cfDNA and matched white blood cells covering 37 lung cancer-related genes	Sensitivity for plasma NGS to detect de novo oncogenic drivers was 75% (68/91) Specificity for plasma NGS in driver-negative tumors compared to tDNA was 100% (19/19)	[66]
Advanced lung adenocarcinoma	Therapeutic planning and serial testing for treatment response, tumor genomic evolution detection	82% of patients had ≥1 ctDNA alteration(s) Concordance for EGFR alterations in ctDNA vs. tDNA was 80.8%; *p* = 0.04	[62]
Carcinomatosis (appendix cancer; colorectal; peritoneal mesothelioma; small bowel; cholangiocarcinoma; ovarian; testicular cancer)	Surgical resection of peritoneal metastases	39% (31/80) of patients had ctDNA alteration Patients with ≥0.25% cfDNA had shorter PFS (7.8 vs. 15.0 months; HR 3.23, 95% CI 1.43–7.28, *p* = 0.005).	[59]
Diverse cancers (including but not limited to: colorectal cancer, non-small cell lung cancer, genitourinary cancers)	*EGFR* amplification status in 28,584 patients	8.5% of diverse cancers had a cctDNA *EGFR* amplification detected Responses were seen in patients with ctDNA EGFR amplification treated with EGFR inhibitors even if no tissue EGFR amplification was detected	[64]
Diverse cancers (including but not limited to gastrointestinal, brain, lung)	Clinical associations of *MET* alterations	7.1% (31/438) and correlated with bone metastasis (*p* = 0.007) *MET* alterations were associated with *TP53* co-alterations (*p* = 0.001) and *PTEN* co-alterations (*p* = 0.003) *MET* alterations were also associated with an increased number of alterations (median, 4 vs. 1, *p* = 0.001)	[65]
Advanced cancers	Ultra-deep cfDNA	Concordance between ctDNA and tDNA NGS was 82–87% Low VAF vs. high VAF of mutant ctDNA had longer OS (*p* = 0.018) Decrease in ctDNA VAF was associated with longer time to treatment failure *p* = 0.03	[67]
Advanced cancers	cfDNA tested with a KRASG12/G13 multiplex assay to detect seven most common mutations in exon 2 hotspot	Concordance was found in 85% (103/121) patients (kappa, 0.66; ddPCR sensitivity, 84%; ddPCR specificity, 88%) Presence of ≥ 6.2% of KRASG12/G13 cfDNA was associated with shorter overall survival (*p* = 0.001)	[71]
Pan-Cancer	Immune checkpoint blockade	Early changes in copy number alterations predicted response versus resistance	[72]

Abbreviations: CI, confidence interval; ctDNA = circulating tumor DNA; DFS, disease-free survival; pCR, pathological complete response; GEJ, gastroesophageal junction; HR, hazard ratio; PFS, progression-free survival; pN+, pathologic node-positive; tDNA = tumor tissue DNA; VAF, variant allele frequency; VUS, variant of unknown significance.
